# Environmental enrichment improves cognitive flexibility in rainbow trout in a visual discrimination task: first insights

**DOI:** 10.3389/fvets.2023.1184296

**Published:** 2023-06-15

**Authors:** Valentin Brunet, Thomas Lafond, Aude Kleiber, Léa Lansade, Ludovic Calandreau, Violaine Colson

**Affiliations:** ^1^Laboratoire de Physiologie et Génomique des Poissons, INRAE, Rennes, France; ^2^Comportement Animal et Systèmes d’Elevage, JUNIA, Lille, France; ^3^Physiologie de la Reproduction et des Comportements, CNRS, IFCE, INRAE, Université de Tours, Nouzilly, France

**Keywords:** cognition, reversal learning, physical enrichment, welfare, *Oncorhynchus mykiss*, operant conditioning

## Abstract

Research on fish cognition provides strong evidence that fish are endowed with high level cognitive skills. However, most studies on cognitive flexibility and generalization abilities, two key adaptive traits for captive animals, focused on model species, and farmed fish received too little attention. Environmental enrichment was shown to improve learning abilities in various fish species, but its influence on cognitive flexibility and generalization abilities is still unknown. We studied farmed rainbow trout (*Oncorhynchus mykiss*) as an aquaculture model to study how environmental enrichment impacts their cognitive abilities. Using an operant conditioning device, allowing the expression of a motivated choice, we measured fish cognitive flexibility with serial reversal learning tests, after a successful acquisition phase based on two colors discrimination (2-alternative forced choice, 2-AFC), and their ability to generalize a rewarded color to any shape. Eight fish were divided into two groups: Condition E (fish reared from fry stages under enriched conditions with plants, rocks and pipes for ~9 months); Condition B (standard barren conditions). Only one fish (condition E) failed in the habituation phase of the device and one fish (condition B) failed in the 2-AFC task. We showed that after a successful acquisition phase in which the fish correctly discriminated two colors, they all succeeded in four reversal learnings, supporting evidence for cognitive flexibility in rainbow trout. They were all successful in the generalization task. Interestingly, fish reared in an enriched environment performed better in the acquisition phase and in the reversal learning (as evidenced by fewer trials needed to reach the learning criterion), but not in the generalization task. We assume that color-based generalization may be a simpler cognitive process than discriminative learning and cognitive flexibility, and does not seem to be influenced by environmental conditions. Given the small number of individuals tested, our results may be considered as first insights into cognitive flexibility in farmed fish using an operant conditioning device, but they pave the way for future studies. We conclude that farming conditions should take into account the cognitive abilities of fish, in particular their cognitive flexibility, by allowing them to live in an enriched environment.

## 1. Introduction

Research on fish cognition provides strong evidence that fish are endowed with high level cognitive skills, ranging from spatial orientation (1), to visual discrimination (2), numerical abilities (3), and conspecific recognition (4). These abilities rely on cognitive functions including perception, learning, memory, and decision-making (5). However, most studies have focused on model species like zebrafish (*Danio rerio*), goldfish (*Carassius auratus*), guppy (*Poecilia reticulata*), or archerfish (*Toxotes chatareus*) (6), and farmed fish have received too little attention so far, especially regarding their cognitive flexibility and their generalization abilities, adaptive traits essential for animals including those maintained in captivity.

Behavioral flexibility is the ability of an individual to adjust its behavior according to changes in the external or internal environment (7). In captive animals, it is a good indicator of their coping ability to a changing or challenging environment (8). In captive fish, the variability of the environment is mostly caused by the unpredictability of stressful events (netting, noises, aggression…), which may lead to poor welfare in the case of low-flexible individuals (9). Fish reared for aquaculture production require flexible behavior to maintain their welfare, adapting to frequent social interactions at high stocking densities, or to unexpected events. Therefore, behavioral flexibility may contribute to reduce stress and maintain fish welfare. This ability is one of the most demanding executive functions (10). Core executive functions include inhibitory control (i.e., the ability to inhibit a routine response), working memory, and cognitive flexibility (8, 10). On the other hand, cognitive flexibility also demands inhibitory control (including focused attention) and working memory. This essential executive function is poorly investigated in farmed fish species. Another important cognitive ability for farmed animals is generalization. Stimulus generalization testing reveals the ability of a subject to use its previous learning when it first encounters new stimuli that physically resemble the stimulus used in initial training (11). The ability to generalize a rule to many stimuli for which the same behavior can be applied enables animals to quickly cope with novelty in their living environment, thereby also reducing the intrinsic stress induced by farming conditions. As far as we know, generalization abilities of visual stimuli have never been assessed in a farmed fish species.

A learning test widely used in model fish species is the two-alternative forced-choice test (2-AFC) in order to study visual discrimination, where two stimuli are presented simultaneously with one being rewarded (2, 12). Cognitive flexibility was assessed experimentally in model fish species by performing a reversal learning test after a successful 2-AFC task. In this case, the stimulus initially rewarded becomes the unrewarded stimulus and the number of sessions needed by the fish to switch and reach the initial learning criteria is measured [zebrafish: (13, 14), guppy: (15), cichlid fish (*Neolamprologus pulcher*): (16)]. Furthermore, by conducting several consecutive reversal tests, we can measure the ability of animals to improve performance across trials. Several setups can be used to evaluate the fish choice in such tests. For example, archerfish have the useful ability to project water onto rewarded targets to indicate their choice, which in this way can be considered as a motivated operant response, making this species an excellent model for studying fish cognition (17). For other fish species for which such precision of choice is not possible, other operant conditioning procedures have been developed to record fish choice as it allows animals to learn that a consequence of a behavior will be followed by either a positive consequence (reinforcer), or a negative one (18). By controlling their behavior, we can assess with a certain confidence that the response of the animal is a motivated choice, as for archerfish. Most of operant conditioning procedures in fish use discrete operant tasks allowing them to indicate their choice when they have to discriminate between two stimuli, such as sensors approach [zebrafish: (14, 19), time spent near stimuli mosquitofish (*Gambusia holbrooki*): (20), zebrafish: (21, 22), and guppy: (23)], or the crossing of a Y-maze [zebrafish: (24)]. However, studies where fish are trained to activate a device, thus exhibiting a motivated choice, are more scarce. Goldman and Shapiro (25) were the first authors to use volunteer self-feeder activation to study cognition in goldfish (25). Based on this pioneer study, our laboratory recently succeeded in using an operant conditioning device to examine visual discrimination in rainbow trout (*Oncorhynchus mykiss*) with a 2-AFC test (26). This was made possible thanks to the correct color vision of rainbow trout. Indeed, they can distinguish at least blue, yellow, red, and green colors (27). While we showed that rainbow trout were able to activate a self-feeder placed in front of reinforced visual stimuli displayed on a screen, thus definitely demonstrating their visual discrimination abilities, we did not examine their cognitive flexibility, or their ability to generalize.

Increasing complexity in captive environments with enrichments improves fish welfare [for reviews (28, 29)], for example by promoting faster recovery from stressful experiences (30, 31). Enrichment, in the form of objects that can be used (such as grapes of leaves or pipes where to hide) or that serves as visual landmarks, provides the animals with more variable sensorial experiences. These experiences provided by enrichment influence brain plasticity as shown by higher neurogenesis and brain cell proliferation in zebrafish (32, 33), and differentially expressed genes and pathways related to cerebral activity, neural plasticity, neurogenesis, and synaptogenesis, in rainbow trout (34). As a result, some studies demonstrated that a complex environment also improves fish cognitive abilities, mainly spatial (35, 36) and social learning (37), including behavioral flexibility when assessed in spatial tasks (38, 39). Moreover, learning abilities are shaped by individual experience during ontogeny and are enhanced when enrichment is provided at early stages (40).

The aim of this study was to investigate the early influence of physical enrichment on rainbow trout’s cognitive flexibility and generalization abilities assessed by visual discrimination tasks using an operant conditioning device (26). We assumed that an operant conditioning procedure would allow for greater accuracy of learned responses since the animal had to make voluntary choices. Rainbow trout is the number one fish species produced in France. Europe being the second largest producer of rainbow trout in the world (41), this gives an idea of the huge quantity of these animals concerned by aquaculture practices and related welfare issues. Rainbow trout were split in two groups: one reared from 99 days post-fertilization (dpf) with physical enrichments for ~9 months and one reared in a barren environment from this stage for ~9 months. Then, we evaluated fish cognitive flexibility by conducting serial reversal learning tasks after an acquisition phase using visual cues (colors) as discriminative stimuli in a 2-AFC test. We hypothesized that rearing fish with environmental enrichments from fry stages specifically improves cognitive flexibility, a poorly investigated executive function in farmed fish, which may be assessed with a non-spatial task. Next, we measured the ability of fish to generalize the last rewarded color to any shape, generalization being a cognitive ability that allows animals to quickly cope with novelty. After assessing whether rainbow trout are flexible and can generalize using an operant conditioning procedure, we hypothesized that rainbow trout reared in an enriched environment from fry stages would perform better than fish previously reared under standard conditions. Together, better cognitive flexibility and generalization abilities would explain the faster ability to recover from stress and the subsequent improved welfare usually observed in enriched fish (28–31).

## 2. Materials and methods

### 2.1. Experimental animals

Female triploids rainbow trout from eggs fertilized at INRAE-PEIMA (Sizun, France) were studied. After being transferred to the Fish Physiology and Genomic Laboratory (LPGP) of INRAE (Rennes, France) at 99 dpf, fish were randomly allocated to two experimental treatments: an early-enriched environment (E) and an early-barren environment (B). The enrichment was composed by PVC pipes, plastic plants (grapes of leaves), and white stones [see (42) for the details about enrichments choice]. A summary of fish mean weight, breeding tank size, number of individuals per tank, number of tanks per treatment, number and types of physical enrichments, as well as estimated floor coverage (%) for each stage until the beginning of testing is presented in Table 1.

**Table 1:** Mean fish weight, tank size, number of individuals, number, type and size of structures, and estimated floor covering at each stage.

Days post-fertilization	Average fish weight (±SEM) at the beginning of each period	Tank size	Number tank replicates and number of individuals	Number (*n*) and type of structures used in the enriched group			Floor covering
				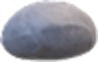	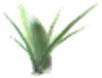	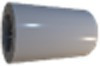	
99–136	2.83 g	72 L (55 cm × 45 cm × 29 cm) Uncovered tanks	Two tanks/treatment 100 individuals each	*n* = 1 (10 cm × 5 cm × 5 cm)	*n* = 2 (24 cm × 15 cm × 11 cm)	*n* = 2(9 cm × 8 cm × 8 cm)	~70%
137–188	11 g		Three tanks/treatment 30 individuals each		*n* = 1 (35 cm × 35 cm × 44 cm)	*n* = 2 (9 cm × 8 cm × 8 cm)	~70%
189–312	32.83 ± 0.67 g	336 L (102,5 cm × 102,5 cm× 32 cm) Covered tanks	One tank/treatment 42 individuals each	*n* = 3 (10 cm × 5 cm × 5 cm)	*n* = 1 (23 cm × 38 cm × 36 cm)	*n* = 1 (11 cm × 9 cm × 6 cm)	~25%
313–364	158.26 ± 5.99 g		One tank/treatment 18 individuals each			*n* = 2 (21 cm × 18 cm × 14 cm)	~40%
						*n* = 1 (19 cm × 14 cm × 28 cm)	
364–673	283.1 ± 5.99 g—1558.9 ± 42.23 g (end of the experiment)	1.8 m^3^ (204 cm × 102 cm × 85 cm) Uncovered tank	20 individuals (10 individuals/treatment grouped in one enriched tank)	/	*n* = 5 (24 cm × 15 cm × 11 cm)	*n* = 3 (21 cm × 21 cm × 22 cm)	~25%
					*n* = 2 (35 cm × 35 cm × 44 cm)		
					*n* = 3 (23 cm × 38 cm × 36 cm)	*n* = 1 (21 cm × 21 cm × 35 cm)	

**Figure 1:**
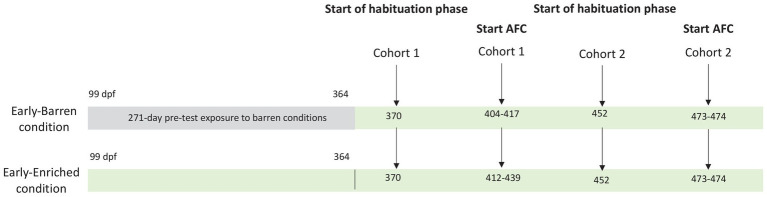
Testing schedule for each condition (early-Barren and early-Enriched) and for each cohort (1 and 2) rainbow trout were distributed. Exposure to enrichment started at 99 days post-fertilization (dpf) in the early-Enriched condition. Both conditions were pooled in the same enriched tank at 364 dpf. Habituation phase to triggers activation started at 370 dpf for cohort 1 and 452 dpf for cohort 2. In cohort 1, the 2-AFC procedure began when fish were between 404 and 435 dpf. Two early-enriched fish (2E) ended the testing procedure by reaching the learning criteria. In cohort 2, the 2-AFC procedure began between 473 and 475 dpf. One early-enriched (1E) and two early-barren fish (2B) ended the testing procedure by reaching the learning criteria.

At 189 dpf (~6.5 months), 4 E and 4 B fish (8 cm mean length) were randomly chosen in the groups and identified with a PIT tag (1.4 mm × 8 mm; Tiny, Biolog-id, France) inserted above the dorsal fin under anesthesia (50 mg/L tricaine). At 364 dpf (~12 months), 20 fish (10 E and 10 B) were pooled in the same enriched environment, including the eight tagged individuals used for cognitive testing. This rearing density was always below 17 kg/m^3^ during the whole experimental period [rainbow trout standards from RSPCA (43)], and the number of 20 individuals allowed to avoid aggressive behaviors and stress due to the hierarchical instability, often observed in groups composed of too few individuals (44). B fish were thus exposed to a barren environment from the fry stage for a period of 271 days (~9 months) before joining E fish in the same enriched tank. Due to the length of testing, fish required to be divided into two cohorts which began their cognitive testing procedures at two different moments making the duration of pre-test exposure to enrichment different between cohorts, as detailed in Figure 1. Nevertheless, each cohort was composed of an equivalent number of E and B fish. For cohort 1, habituation phase for testing started at 370 dpf, while for cohort 2, habituation phase started at 452 dpf. Pooled fish from both conditions were all exposed to the same enriched environment while tested. Therefore, B fish from cohort 2 had been exposed to enrichments for ~3 months before starting the habituation phase, while enrichment exposure lasted only 6 days for B fish from cohort 1.

All breeding and test tanks were supplied by circulating and recycled water maintained at 12 ± 0.2°C. The water quality was regularly checked (NH4 +, NO2 −, NO3 −). The artificial photoperiod was 12:12 h. Fish were fed daily at 16:00 (when the experiments ended) with extruded and commercial flowing extruded pellets (39% protein and 24% lipid, Le Gouessant, France). Diameter of the pellets and feeding rate were regularly adapted according to the fish growth weight, as detailed in our previous study (42).

### 2.2. Experimental device

**Figure 2:**
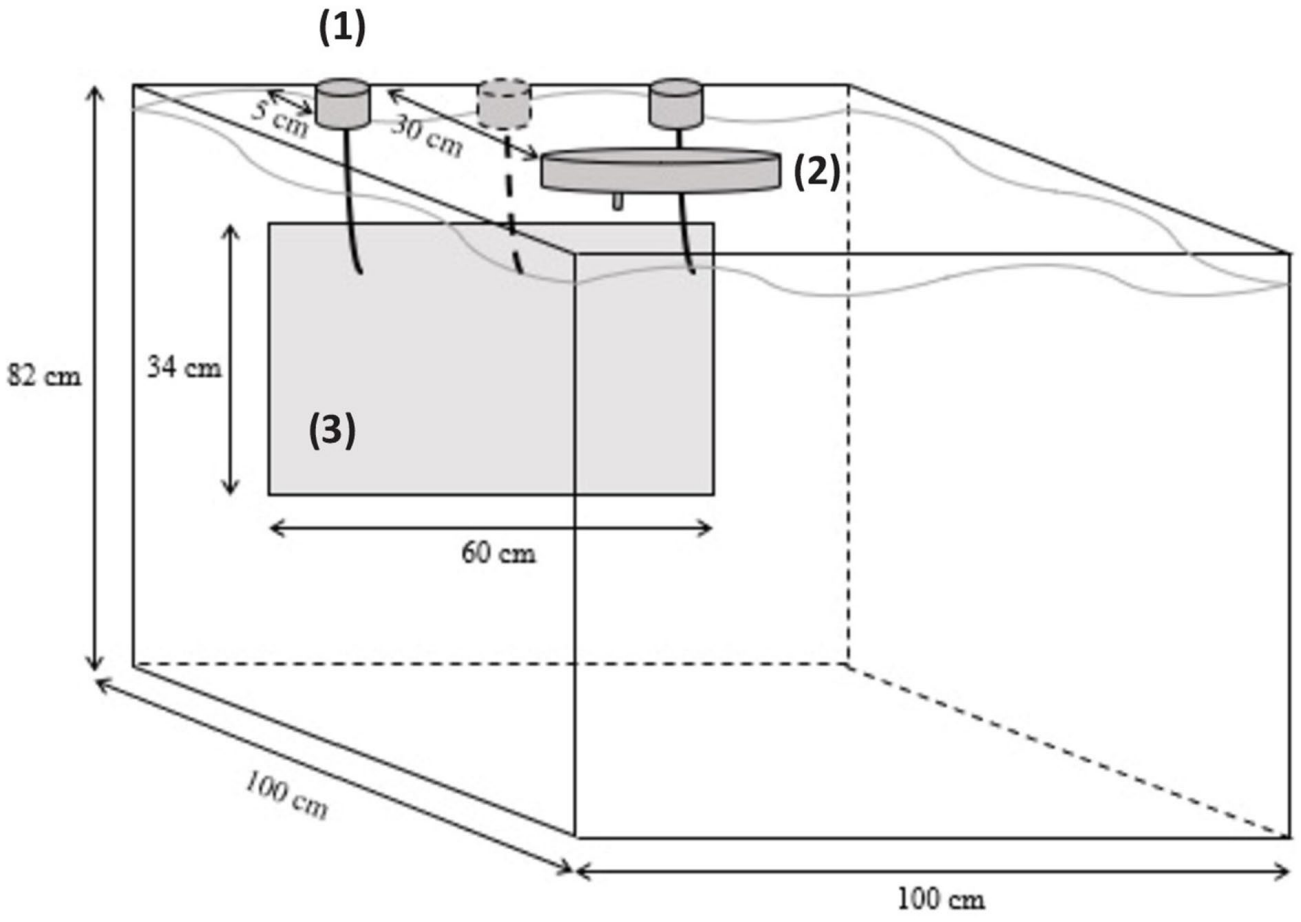
Experimental tank used for cognitive testing procedures. (1) Self-feeders (one of the three being removable); (2) Food distribution wheel; (3) Transparent Plexiglas window behind which visual stimuli were displayed on a screen.

The experimental device was the same as in Kleiber et al. (26). Fish were individually introduced into a 779-L test tank (104 cm × 100.5 cm × 74.5 cm; Figure 2). A transparent glass (63 cm× 34 cm) was insert into the front side of the tank, allowing fish to see a screen (LCD Dell REV A00) placed on the other side. When switched on, the screen continuously displayed a light gray color (RGB code = #ECECEC) so as not to denote the walls of the tank. Self-feeders’ triggers dived to 9 cm from the surface and 5 cm from the screen. They could be activated by the fish with a pressure (5–6° angle) on it. When fish activated the trigger facing the positive stimulus, a food reward was released in the middle of the test tank (approximately at 30 cm from the screen), by an automatic food distribution wheel (Imetronic®, France). The food reward represented 1/12 of the daily food ration and was adapted to the fish growth. If the incorrect trigger was activated, no food reward was distributed. Tests were managed by the software “POLY M2S” (Imetronic®), which allows the experimenter to choose the type of test (habituation, 2-AFC, reversal learning or generalization), the number of trials, the inter-trial interval (ITI), the different visual stimuli to display, and their position on the screen. A video camera was placed above the test tank and was linked to a monitor to control the experiment without disturbing fish.

### 2.3. Testing procedures

#### 2.3.1. Habituation phase

**Figure 3:**
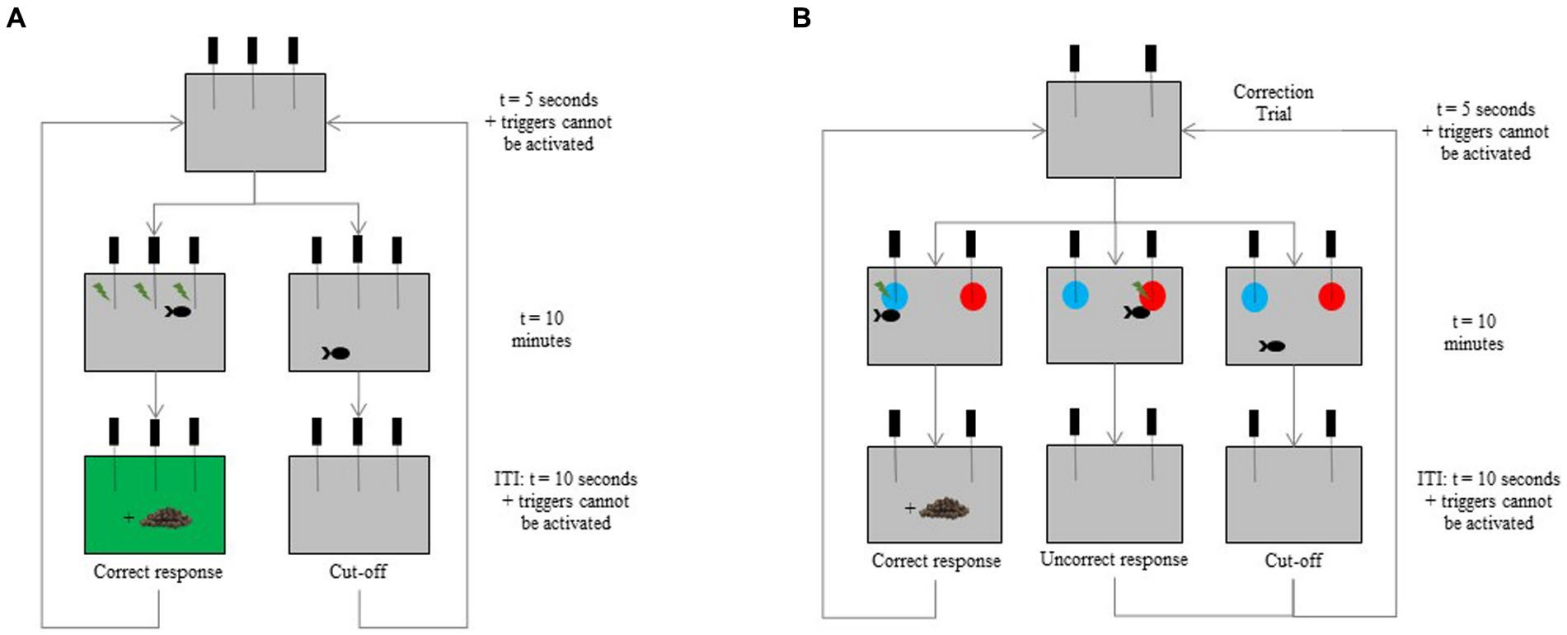
Schematic view of the successive screens during **(A)** the device habituation where three triggers are available here, and **(B)** the visual discrimination tasks where only two triggers remain. The two discriminative stimuli (blue and red disks) are presented in the top corners left and right of the screen. In this example, the fish activates the trigger positioned in front of the blue disk to obtain a food reward. A correction trial is implemented in the case of cut-off or incorrect response, reusing the exact same set of stimuli until a correct response is made.

The eight tagged fish (4E and 4B) were trained to use the device. At their first entrance in the test tank, a food reward was provided. Fish were then trained to activate two or three triggers (depending on the learning difficulty of each individual) to obtain a reward (Figure 3A). The food reward was accompanied by a green screen (RGB code = #0F790) during 10 s which acted as a secondary reinforcing stimulus. Each fish had one session per day, and training days were not systematically consecutive. The first session was composed of five trials of 10 min each for the first session, and 10 trials of 10 min for the following sessions. Each trial began with 5 s during which the triggers could not be activated. If the fish did not activate a trigger within 10 min, the response was recorded as a “cut-off.” At the end of a trial, a new trial automatically began after an ITI of 10 s where triggers could not be activated. Individuals had to activate the triggers at least seven times over the 10 trials in three consecutive sessions (≥70%; binomial: *p* < 0.001, *N* = 30 trials) to meet the learning criterion and to move on the following stage. The decision to exclude an individual from the exercise was made once the frequency of trigger activation was less than 30% over 10 consecutive sessions. After each session, the fish was gently netted and joined its congeners in the breeding tank.

#### 2.3.2. Visual discrimination

From this phase, only two triggers were available, one being rewarded (right or left position; Figure 3B). Because one individual (#6241 from condition E, see Table 2) failed to activate the device during the habituation phase, and because of a technical problem when starting the acquisition phase for one individual from condition B (#0977), only six individuals (3E and 3B) were tested in the subsequent tasks.

**Table 2:** Total number of sessions needed for each individual to reach the learning criterion during each learning task.

	Habituation	2-AFC	Reversal 1	Reversal 2	Reversal 3	Reversal4	Generalization 1	Generalization 2	Generalization 3	Generalization 4
**Early-enriched**	
*#6241*	10									
*#6224*	6	3	6	3	3	4	2	2	2	2
*#6246*	12	6	3	3	2	4	2	2	2	2
*#6250*	7	5	18	5	3	4	4	2	2	2
**Early-Barren**
*#0624*	6	8	9	5	5	4	3	2	2	2
*#0647*	9	10								
*#0977*	12									
*#6228*	6	20	4	5	8	6	2	2	2	3

##### 2.3.2.1. Acquisition phase with a 2-alternative forced choice test

In the acquisition phase, fish were trained to push the respective triggers positioned in front of a blue (RGB code = #1E90FF) or a red (RGB code = #FF0000) disk (Ø = 11 cm) displayed on the screen. The rewarded color (positive stimulus, S+) was randomly distributed, so that three E fish and three B fish were trained either on the blue or on the red. In the test tank, fish had the choice between two discriminative stimuli (one behind each trigger) in the top corners left and right of the screen (Figure 3B). Side presentation was randomly counterbalanced, and the S+ never appeared more than twice consecutively on the same side. In case of a correct response within 10 min, the fish received the food reward and the next trial automatically began after an ITI of 10 s. If the incorrect trigger was activated, no food reward was distributed, and a 10-s ITI was run before starting a correction trial (CT) to prevent any side bias, using the same procedure as described in Knolle et al. (45). The CT was implemented in the case of an incorrect response or a cut-off (no trigger activated) (45), reusing the exact same set of stimuli until a correct response was made. Then, after a correct response in a CT, a classical trial could start again (Figure 3B). A session was composed of 12 classical trials. Individuals had to select the S+ at least nine times over the 12 classical trials in two consecutive sessions (≥75%; binomial: *p* = 0.011, *N* = 24 trials) to meet the learning criterion and to move on the following stage. The decision to exclude an individual from the exercise was made once the frequency of trigger activation or of correct responses was less than 30% over a maximum of 10 consecutive sessions.

##### 2.3.2.2. Serial reversal learning

After fish succeeded in the acquisition phase with the 2-AFC, we performed four consecutive reversal learning tasks to study their cognitive flexibility. For example, after a fish passed the acquisition phase with the blue disk as S+, the red disk became the S+ in the reversal 1, and so on until reversal 4 (S+ was a blue disk again). The same parameters as for the acquisition phase were used. For each reversal task, a session was composed of 12 classical trials. The learning criterion for moving to the next stage of reversal was set at 75% of correct responses in classical trials on two consecutive sessions (≥ 75%; binomial: *p* = 0.011, *N* = 24 trials). When the learning criterion was reached, an additional session was run to consolidate the associative learning of the last rewarded color before moving to the generalization task.

##### 2.3.2.3. Generalization task

**Figure 4:**
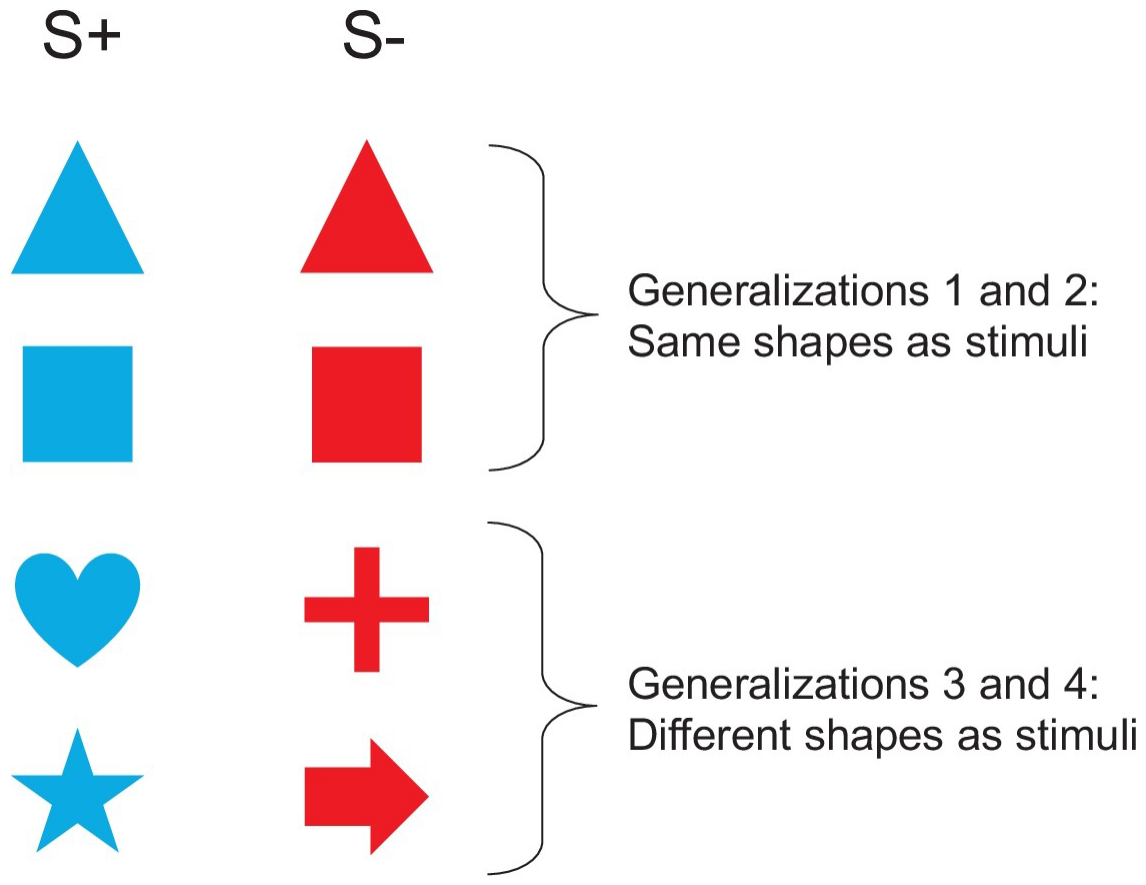
Stimuli used in the generalization task. In this example, the blue shapes are rewarded.

After the reversal learning, we performed four consecutive generalization tasks to assess the ability of fish to generalize the last rewarded color to any shape. For example, an individual that completed reversal 4 with a blue disk as S+, was assessed for its ability to generalize the rule “the color blue is rewarded” to any shape. In this case, the fish was rewarded once the trigger placed in front of the blue shape was activated. For generalization tasks 1 and 2, the two shapes to discriminate on the basis of their color were similar (but different from the disk used in the reversal learning task), but difficulty increased in generalizations 3 and 4 where the two shapes were different (Figure 4). We first presented two triangles (one blue and one red, generalization 1), then two squares (generalization 2), then one heart *vs* one cross (generalization 3), and finally one star *vs* one arrow (generalization 4). The shapes were all 95 cm^2^ in area. The same parameters as for the previous tasks were used. For each generalization task, a session was composed of 12 classical trials. The learning criterion was set at 75% of correct responses in classical trials on two consecutive sessions (≥ 75%; binomial: *p* = 0.011, *N* = 24 trials).

For each task, we calculated the total number of classical trials (excluding CT) and the number of sessions (for habituation phase) needed for each individual to reach the learning criteria as previously defined, as well as the mean percentage of correct responses per session (excluding CT). The software POLY M2S automatically calculated others variables of interest: the total number of cut-offs as trials, and the latency before the first correct response was given (minutes). This latency was the period of time between the trial start and the correct trigger was first activated and reflected the achievement of the task in a given amount of time: the faster the task was performed, the lower the latency.

### 2.4. Statistical analyses

All analyses were performed with the R © software version 4.2.0. We used the following packages: “car” to calculate the ANOVA tables, “MASS,” “lme4,” and “nlme” for the mixed models and “emmeans” for the post-hoc tests. The diagrams were created with the “ggplot2” package, except for numbers of trials with or without cut-offs which were compiled in an Excel diagram.

At the individual level, binomial tests were conducted to assess the statistical significance of performance above chance over two consecutive sessions (three for the habituation phase) which determined the learning criterion for fish to proceed to the next exercise (see specific paragraphs in the section Materials and methods).

For the habituation phase, the number of sessions (and the total number of classical trials) needed to reach the learning criterion were compared beween the two treatments (E and B) using a non-parametric Wilcoxon test (unpaired data specified).

The global effects of cohort (cohort 1 or 2) and stimulus color (red or blue) were also assessed. For each task, we found no effect of cohort or stimulus color on any of the variables of interest, therefore these factors were excluded from each model.

The number of classical trials (excluding CT) needed to reach the learning criterion was analyzed with a Generalized Linear Mixed Model (GLMM) with “lme4” package, since the dataset followed a Poisson distribution. The following model was used:


$$\mathrm{glmer}\;\left(\begin{array}{l} \mathrm{Nb}\;\mathrm{trials}\sim {\mathrm{Condition}}^{*}\;\mathrm{Task}+\left(1\mathrm{|individual}\right),\;\\ \mathrm{family}=\mathrm{poisson}\left(\mathrm{link}=“\log”\right) \end{array}\right)$$

The mean percentage of correct responses (excluding CT), and the latency before the first correct response were analyzed using ANOVAs (type III) for repeated measures since the dataset followed a normal distribution after asin-and log-data transformations, respectively. Mixed Linear Models (LMM) were run with “nlme” package, as follows:


$$\mathrm{lme}\;\left(\mathrm{asin}\left(\%\mathrm{CR}\right)\sim {\mathrm{Condition}}^{*}\;\mathrm{Task, random}=\sim 1\mathrm{,|individual}\right)$$

$$\mathrm{lme}\;\left(\log\left(\mathrm{LatenceCR}\right)\sim {\mathrm{Condition}}^{*}\;\mathrm{Task, random}=\sim 1\mathrm{,|individual}\right)$$

For each model, Condition (E and B) and Task (2-AFC, reversals 1–4, and generalizations 1–4) were the fixed explanatory factors, and individuals were considered as random factors in order to take into account the dependence structure in the data.

For the global analysis comparing all tasks, the small sample size prevented to include the conditions into the model. Therefore, results were analyzed independently of the conditions to assess global fish learning performance in serial reversal learning and in generalization tasks. The model was run as follows:


$$\mathrm{glmer}\;\left(\begin{array}{l} \mathrm{Nb}\;\mathrm{trials}\sim \mathrm{Task}+\left(1\mathrm{|individual}\right),\\ \mathrm{family}=\mathrm{poisson}\left(\mathrm{link}{=}^{“}\log^{”}\right) \end{array}\right)$$

*Post hoc* tests of multiple comparisons were performed with the “emmeans” package if the interaction Condition x Task was significant (*p* < 0.05).

The global effects of cohort (cohort 1 or 2) and stimulus color (red or blue) were also assessed. For each task, we found no effect of cohort (cohort 1 or 2) or stimulus color (red or blue) on any of the variables of interest, therefore these factors were excluded from each model.

During the 2-AFC task, the number of trials (excluding CT) with and without cut-offs were calculated in conditions E and B. Differences between observed and theoretical numbers of trials were statistically compared using a chi-square test.

*p* values <0.05 were considered statistically significant for all analyses.

## 3. Results

### 3.1. Habituation phase

Of the eight fish included in this experiment, one failed in the habituation phase displaying no motivation to activate the triggers (the frequency of activation of the triggers was less than 20% over 10 consecutive sessions), and was excluded from subsequent tasks (Table 2; Supplementary Figure S1). This fish (#6241) belonged to condition E. The mean (+/− SEM) number of sessions needed to reach the learning criteria for habituation phase was 8.75 +/− 1.38 in condition E and 8.25 +/− 1.44 in condition B (i.e., 82.5 +/− 13.77 classical trials needed in E and 77.5 +/− 14.36 trials in B), which was not significantly different (Wilcoxon test: W = 6.5, value of *p* = 1).

### 3.2. Visual discrimination

#### 3.2.1. Analysis of the acquisition phase with a 2-AFC

One fish (#0977) from condition B was discarded because of a technical issue when starting the acquisition phase.The learning criterion was reached for five out of the six fish tested in the acquisition phase, with the S+ selected on nine (or more) of the 12 classical trials (excluding cut-offs) during two consecutive sessions (≥ 75%; binomial: *p* = 0.011, *N* = 24 trials). E fish needed three, six, and five sessions, respectively, to reach the criterion and B fish needed 8 and 20 sessions, respectively (Table 2; Supplementary Figure S1). The B fish (#0647) which failed in this task spent 10 sessions not activating the triggers or giving correct responses in less than 20% of the trials. It was thus excluded from the subsequent tasks.

**Figure 5:**
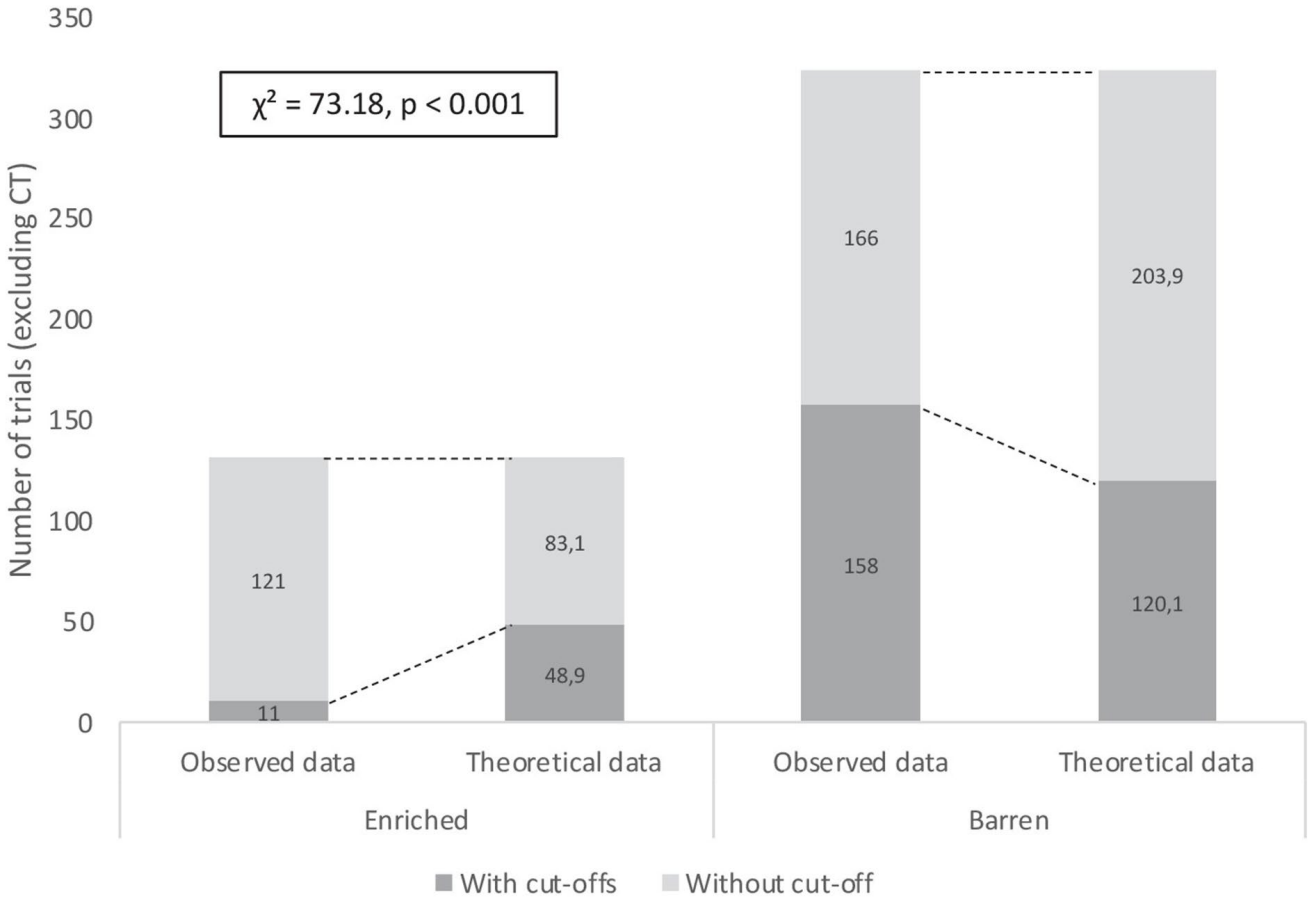
Numbers of classical trials (excluding correction trials) with and without cut-offs during the 2-AFC acquisition phase performed by rainbow trout from early-Enriched and early-Barren conditions. Differences between observed and theoretical numbers of trials were highly significant (Khi-2 test, *p* < 0.001).

In the 2-AFC acquisition phase, E fish exhibited fewer cut-offs (no trigger activated during a classical trial) than B fish. The chi-square test showed a highly significant difference between the observed numbers of classical trials with cut-offs in conditions E (11/132 trials) and B (158/324 trials) and the calculated theoretical numbers of trials with cut-offs (E: 48.9/132 trials; B: 120.1/324 trials; χ^2^ = 73.18, *p* < 0.001; Figure 5).

#### 3.2.2. Analysis of the procedure including the acquisition phase (2-AFC), followed by four serial reversal learning tasks

All the remaining fish (3 E and 2 B) successfully performed in the serial reversal learning task in less than 18 sessions (Table 2).

**Figure 6:**
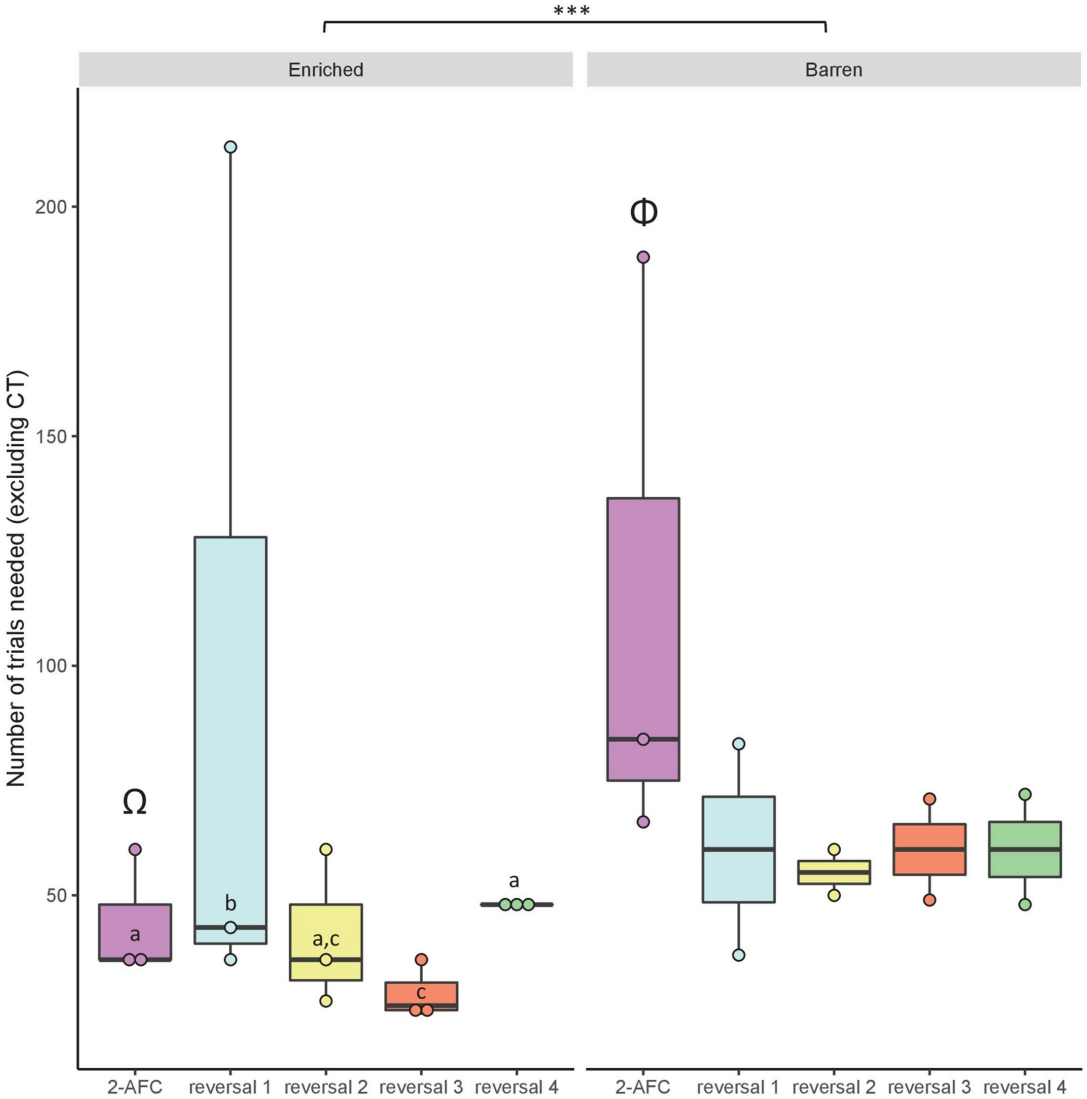
Median (quartiles: 25 and 75%) number of classical trials (excluding correction trials) needed for the fish from early-Enriched (E) and early-Barren (B) conditions to reach the learning criterion in the visual discrimination tasks: 2-AFC acquisition phase, reversals 1–4. Differences between conditions are represented by ^***^ (GLMM: *p* < 0.001). *Post hoc* tests have been run and differences between tasks within condition E only are represented by different latin letters (*p* < 0.001). For the reversal tasks, statistical analyses were not run within condition B because of low sample size. Two different Greek capitals represent a statistical difference between conditions E and B within the 2-AFC acquisition phase (*p* < 0.001).

When considering the number of classical trials needed, we found a significant interaction between condition and task factors (GLMM: χ^2^ = 111.39, df = 4, *p* < 0.001). There was a significant effect of the fixed factor condition (χ^2^ = 12.29, df = 1, *p* < 0.001) and task (χ^2^ = 150.94, df = 4, *p* < 0.001). Fish from condition B needed more trials than E fish to reach the learning criterion (mean ± SEM: B: 73.54 ± 12.40 trials vs. E: 51.8 ± 11.86 trials, *p* < 0.001) in the 2-AFC task (*p* < 0.001; Figure 6). *Post hoc* tests also revealed that within condition E, fish needed more trials to achieve the first reversal (97.33 ± 57.87 trials) than the 2-AFC task (44 ± 8 trials; *p* < 0.001) and reversal 3 (28.67 ± 3.71 trials; *p* < 0.001). The contrary was observed within condition B where fish needed more trials to perform in the 2-AFC task (112.99 ± 38.36 trials) than in the successive reversals (Figure 6). However, these comparisons within condition B are only descriptive due to the small number of remaining fish.

For this procedure including the 2-AFC and the serial reversals tasks, we found no significant effects of condition (GLM: *F*_1,4_ = 0.006, *p* = 0.941) and task (*F*_4,12_ = 0.468, *p* = 0.758), nor significant interaction between these two factors (*F*_4,12_ = 0.913, *p* = 0.487) on the latency before the first correct response.

#### 3.2.3. Analysis of the generalization tasks

All the fish successfully performed in the generalization tasks in less than four sessions (Table 2; Supplementary Figure S1).

When including in the model only the generalization tasks, we found no significant effect of the two factors: condition (*p* > 0.05) and number of the generalization tasks (*p* > 0.05) on any of the variables of interest.

#### 3.2.4. Comparisons of the different tasks (from 2-AFC to generalization 4)

**Figure 7:**
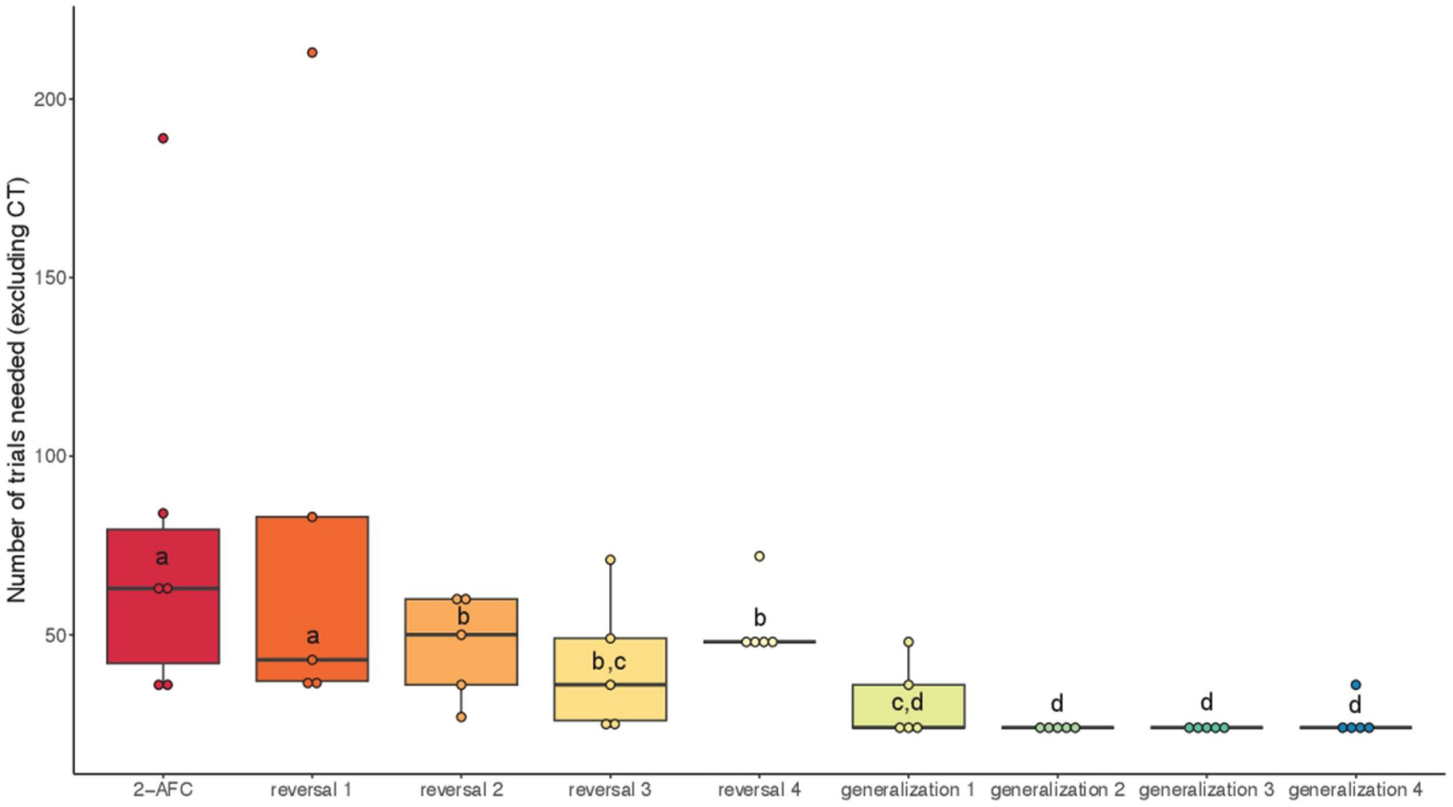
Median (quartiles: 25 and 75%) number of classical trials (excluding correction trials) in the successive visual discrimination tasks (conditions pooled): 2-AFC acquisition phase, reversals 1–4 and generalizations 1–4. Differences between tasks are represented by different letters (*p* < 0.01).

When considering all the tasks performed by the fish and considering the number of classical trials needed to reach the criterion independently of the condition, we found a significant effect of the task (GLMM: χ^2^ = 407.72, df = 8, *p* < 0.001). *Post hoc* tests revealed that fish took longer to reach the learning criterion in 2-AFC and reversal 1 tasks compared to reversals 2–4 (*p* < 0.001; Figure 7). Regarding generalizations, fish were faster to reach the criterion in generalizations 1–4 compared to 2-AFC and reversals 1, 2, and 4 (*p* < 0.01; Figure 7).

**Figure 8:**
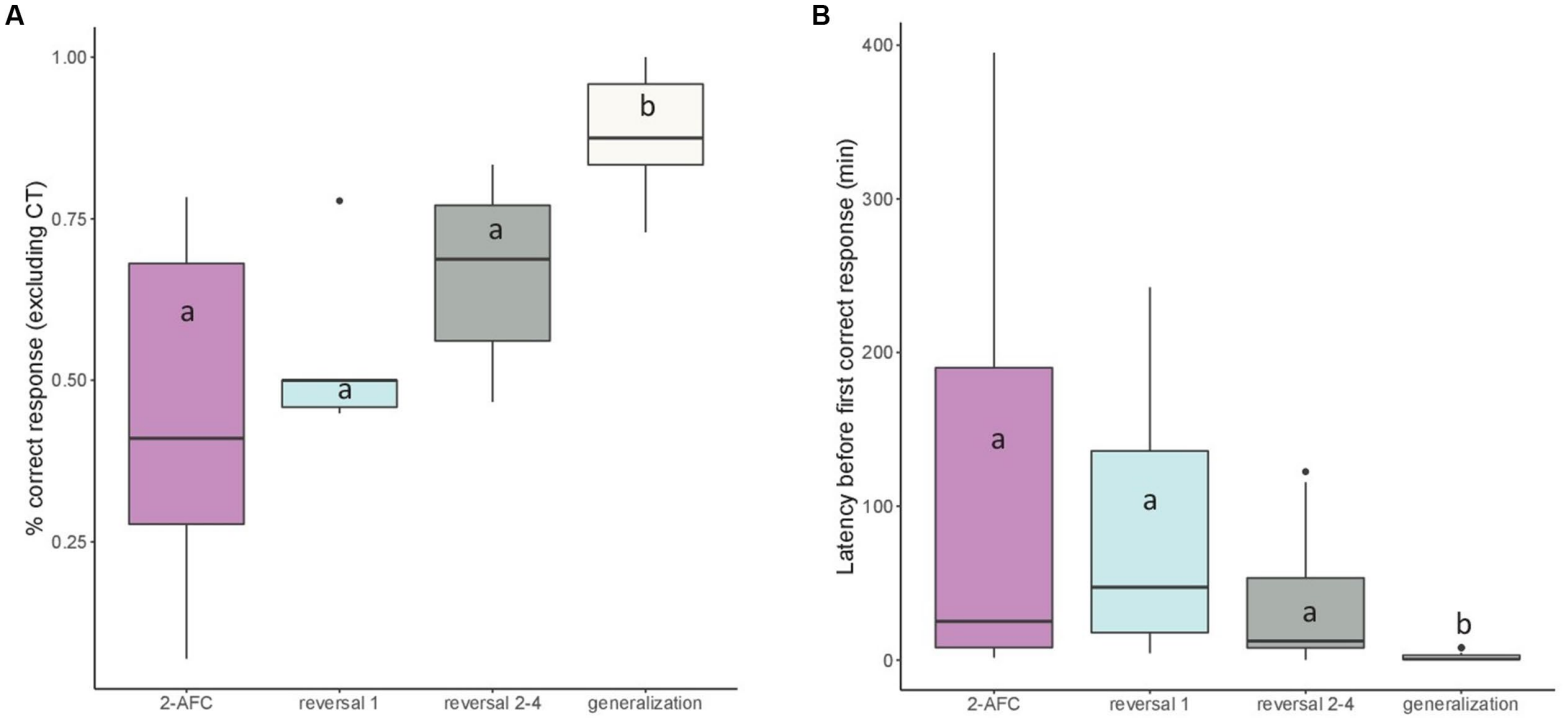
Median (quartiles: 25 and 75%) **(A)** percentage of correct responses per session (excluding correction trials) and **(B)** latency before performing a first correct response (min) in the successive visual discrimination tasks: 2-AFC acquisition phase, reversal 1, reversals 2–4 (pooled), and generalizations 1–4 (pooled). Differences between tasks are represented by different letters (*p* < 0.05).

Considering the mean percentage of correct responses given in classical trials per session, we found that this percentage was higher in E (mean ± SEM: 78.08 ± 3.42%) than in B (64.13 ± 5.27%, *p* = 0.019). It was also higher during the generalization tasks than during the others previous tasks (*p* < 0.05; Figure 8A).

Considering the latency before performing a first correct response, we found a significant effect of the task on this variable (*F*_3,37_ = 14.64, *p* < 0.001). *Post hoc* tests showed that this latency was lower in generalization tasks, indicating that fish were faster to give their first correct response in generalization tasks than in other tasks (*p* < 0.001; Figure 8B).

## 4. Discussion

As previously shown in Kleiber et al. (26), rainbow trout were perfectly able to use an operant conditioning device with concrete responses (volunteer self-feeder activation) allowing a decision-making process when submitted to visual discrimination tasks. In this study, rainbow trout were able to discriminate blue and red circles in order to get a food reward, to modify their response to a contrary rule, and to generalize to any shape the last rewarded color. As hypothesized, fish performed better when reared in an enriched environment from early life stages.

Rainbow trout successfully performed the acquisition phase (2-AFC task), reaching the learning criterion in only 44 ± 8 trials in average (± SEM) in condition E, and in 112.99 ± 38.36 trials in condition B. This performance is almost similar to our previous results where rainbow trout needed 84–168 trials to discriminate between photographs of conspecifics and a black shape (26). As a comparison, archerfish needed 30–90 trials to discriminate between orange and purple circles on a screen (17). By extending to other vertebrates, horses needed 80 trials on average to discriminate between two 2-D stimuli displayed on a screen (46), and chicken needed 300 trials to reach the learning criterion in a similar visual discrimination task using a screen-like apparatus (47), making the visual discrimination abilities of rainbow trout broadly equivalent to those of phylogenetically distant vertebrates. Note that this conclusion relies on only six individuals available for this experiment. In learning procedures, when cognitive abilities are demonstrated in a few individuals [only three in the study performed on horses for example (46)], it is considered that these abilities may be generalized to the whole species meaning that the brain of all the members of the species is endowed with neurocognitive systems that likely support the resolution of the task (48). However, our findings should be confirmed in future studies with larger numbers of animals.

An individual’s cognitive flexibility is its ability to switch from one cognitive task to another (49). Here, rainbow trout were able to modify their operant conditioned response in case of a shift in reward contingencies, extinguishing the previously reinforced behavior and responding to the new contingency in the serial reversals which followed the initial acquisition phase. The first reversal was completed after a minimum of 36 trials and a maximum of 213 trials by the five remaining individuals at this stage. Then, the interest of serial reversal relies on measuring an improvement in learning through experience, requiring to be increasingly fast to update learnt associations or learn new ones as the rewarding value of the visual stimuli change through time. After the first reversal, we observed this improvement in rainbow trout, which completed the subsequent 2–4 reversals in rapid succession in only 24 trials (for the fastest individual) to 72 trials (for the slowest), suggesting that fish learned to learn that the rule could change, with a likely threshold in the number of trials after the second reversal in our experiment. The improvement in performance over successive reversals has been evaluated within a large number of animal species including primates (50), rodents (51), avian species (52), and bumblebees (53). Cognitive flexibility were more scarcely studied in fish species. In rainbow trout (39) and Atlantic salmon (*Salmo salar*) (35), behavioral flexibility has been observed but in spatial reversal learning tasks, where fish had to switch to a new food source. However, spatial relocations are not necessarily equivalent to reversal learning of nonspatial discrimination, which involves “unlearning” an old association before forming a new one (8). Here we show for the first time that rainbow trout is capable of cognitive flexibility in a context other than spatial cognition, namely in a visual discrimination task using a two-lever operant procedure where the response-outcome association is reversed. Again, more individuals would be needed to support this result, but the statistical evidence despite the small number of fish tested is promising. The flexible behavior displayed by rainbow trout suggests not only motivated choice by voluntarily activating a trigger [this is not a simple discrete response, as usually observed with sensors or stimuli approach in fish cognition studies (19, 23)], but also complex cognitive functions. These functions range from associative learning and temporal association of environmental cues required in operant conditioning (54), to inhibitory control (including focused attention) (8, 55) and working memory, two other executive functions (10). What is difficult when processing cognitive flexiblility is to move from one mental rule to another making this skill one of the most demanding executive function. To do so, some individuals needed only two sessions. These results provide first insights into the application of such operant conditioning procedure to further investigate cognitive flexibility on fish species of commercial interest.

Generalizing the last rewarded color to any shape appeared to be easily acquired by all individuals regardless of their rearing condition, as evidenced by the minimal number of trials required to reach the learning criterion, the high percentage of correct responses and the minimal latency before responding correctly, compared to all previous tasks. In general, decreased in latencies to respond indicates learning acquisition process (56) or motivation to access a reward (57). The high performance obtained by all individuals induced a ceiling effect, preventing any discrimination between the two experimental conditions, which seemed to have no impact on generalization abilities. Another explanation, in addition to that concerning the small number of individuals remaining at this stage, could be that unlike cognitive flexibility, which is one of the most demanding of the executive functions (10), color-based generalization can be performed in a more automatic way (same color regardless of the shape—response), which may represent a less focused-attention task. In triggerfish (*Rhinecanthus aculeatus*), discriminative learning relies on color of the stimulus rather than on the pattern (58). This supports that our color-based generalization task may be fairly simple for fish, especially since they had an additional session at the end of reversal 4 to consolidate the color-reward association. Therefore, the complexity of the environment could have a positive influence on certain fish cognitive abilities that require a particular attentional set (e.g., visual discriminative learning and flexibility) (8), but not on other simpler cognitive functions such as generalization based on an attended color. Being able to generalize a rule to many stimuli that share a same feature and for which the same behavior can be applied allows animals to quickly make decision regardless of their level of attention, and to quickly cope with novelty in their habitat, hence reducing stress. Considering the present color-based testing procedure, this ability seems very pronounced in rainbow trout even if they were reared under barren conditions at early stages. A generalization task for which fish have to process a same shape regardless of the color would be an interesting opposite procedure to complete the cognitive panel of rainbow trout. This would have implied that the initial acquisition phase rewarded the shape and not the color. Further experiments would be necessary to definitively determine the generalization abilities of rainbow trout regardless of the features of the stimuli.

Fish reared under an enriched environment from early life stages were faster to reach the learning criterion and gave more correct responses than B fish when pooling the acquisition phase and the serial reversal learnings. The E fish, which completed the acquisition phase in only 44 trials in average, needed then 97.33 trials in average to achieve reversal 1. This is in line with what is usually observed in cognitive flexibility studies carried out on other species, where learning performance usually decreases at the first reversal learning stage in comparison with the acquisition phase (47, 59). On the other hand, B fish took longer to complete the 2-AFC task than E fish, and one B fish did not succeed while already habituated to the apparatus, suggesting a potential effect of living conditions on fish emotional reactivity, as we observed in another experiment (42). However, once the initial visual discrimination was acquired, serial reversals were completed quickly in a few trials, even the first reversal (from descriptive-only results). Therefore, for B fish, we did not observe the decrease in performance often shown at reversal 1, that we observed in E fish. This may be due to the learning performance in the acquisition phase, which was already low in B fish compared to E fish (112.99 vs. 44 trials needed in average, respectively). All together, when pooling the acquisition phase and reversal learnings, our results are consistent with the literature, which is encouraging, since environmental enrichment, or a varying environment, has already been shown to improve the cognitive abilities of farmed fish (35, 37). Flexibility of feeding behavior is also enhanced by physical enrichment as seen in fish ability to switch to a new food source (60), or to find novel paths to gain access to food (39), faster than fish reared under barren conditions. Our results suggest that increasing the complexity of the environment from fry stages not only promotes better learning performance in rainbow trout, but also improves their cognitive flexibility in a visual discrimination task, a context other than spatial cognition.

We did not find any effect of the cohort although some B fish from cohort 2 were sometimes exposed to environmental enrichment for 3 months before being tested. The major difference between early-barren and early-enriched conditions was that early-enriched fish were exposed to enrichment for 9 (cohort 1) to 12 months (cohort 2) before being tested, while early-barren fish could be exposed for 0 (cohort 1) to 3 months (cohort 2) at an advanced age (i.e., 473 dpf ~12.4 months). In condition E, this 9-month exposure from early stages, which is a long period comparing to classical enrichment studies, could have revealed differences in cognitive flexibility between treatments (despite the small samples size), which might be hidden when exposure to enrichment is shorter [1 month in guppies (23)]. Furthermore, the lack of difference between cohorts and the lack of effect of three-month enrichment exposure at an advanced age in B fish confirms the phenotypic plasticity induced by an early experience (40, 61). Cognitive flexibility is considered as an essential adaptive trait obviously in wild animals for facilitating mate choice, foraging or predator/prey detection (62), but also for fish living in captivity. Farmed fish require flexible behavior to adapt to frequent social interactions at high stocking densities. For example, individual recognition of numerous conspecifics and flexible memory of their hierarchical position saves energy in cognitive demand and stress response (63). Furthermore, unexpected events occurring under farming conditions may be better managed by fish endowed with a suitable behavioral flexibility, by preventing the stress associated with intrinsic unpredictable farming conditions (9). Therefore, our results suggest that providing captive juvenile rainbow trout with the opportunity to live in a complex environment enhances their cognitive flexibility later in life, which may help them better cope with sudden, unexpected and stressful events, thereby promoting their welfare.

Interestingly, we found that fish from condition E performed significantly more trials without cut-off than trials including cut-off compared to B fish during the 2-AFC acquisition phase. Since trials including cut-off represent an absence of response (self-feeders were not activated during the trial), it may suggest that fish were no longer motivated to obtain the food-reward, or more likely that the 2-AFC condition, where visual stimuli appeared on the screen for the first time, was considered as a stressful event for B fish. Moreover, the only fish which failed in this task, spending 10 sessions not activating the triggers or giving correct responses in less than 20% of the trials was a fish from condition B. Anxiety was shown to decrease in fish reared in enriched environments [Atlantic cod (60)]. As also shown in Brunet et al. (42), rainbow trout reared in an enriched environment exhibited fewer anxiety-related behaviors in social isolation and were more curious when confronted with a novel object. Here, the first display of visual stimuli on the screen during the acquisition phase can be likened to a novel-object test, and could explain the increased absence of response in B fish. Moreover, we previously found that environmental effects on anxiety-like behavior were associated with differencially expressed genes (neurotrophic, neurogenesis, synaptogenesis markers, and genes associated with dopaminergic and serotonergic systems), essentially located in the telencephalon, the fish brain area involved in memory, learning and emotions (34). Recent evidence in mammals suggests a prominent role of dopamine and serotonin in cognitive processes, in particular reversal learning (55, 64). If providing fish with a complex environment can modulate the expression of genes involved in these neurotransmitter systems (34), the improvement in reversal learning that we observed in rainbow trout reared in an enriched environment from early stages would make sense, but deserves further studies in fish species with larger numbers of individuals.

## 5. Conclusion

Our study illustrates how standard operant conditioning approaches can be used to explore the cognitive abilities of a non-model fish species. Despite the small number of individuals we used, we showed that rainbow trout, one of the major farmed fish species in Europe, is capable of cognitive flexibility when tested in a nonspatial task (i.e., a visual discrimination task using an operant conditioning device), suggesting motivate choice and complex cognitive functions. Moreover, rainbow trout reared in a complex environment from fry stages had not only better learning performance in the initial acquisition phase as evidenced by fewer trials needed to reach the learning criterion than fish previously reared in a barren environment, but also better cognitive flexibility in the serial reversal learning. Providing physical structures in the environment of juvenile captive fish may promote appropriate cognitive flexibility later in life that allows fish to better adapt to stressors associated with intrinsic husbandry conditions, thereby helping to maintain fish welfare. Interestingly, the ability to generalize the last rewarded color to any shape seems very pronounced in rainbow trout even if they were previously reared under barren conditions. This can be ascribed to the fact that generalization based on an attended color is a simple cognitive process that does not seem to be influenced by environmental conditions. We conclude that farming conditions should take into account the cognitive abilities of fish, in particular their cognitive flexibility, by allowing them to live in a complex environment from early stages, which would encourage behavioral plasticity, given the strong relationship between environmental stimulations, cognition, and welfare.

## Data availability statement

The raw data supporting the conclusions of this article will be made available by the authors, without undue reservation.

## Ethics statement

All experimental procedures were performed under the European directive 2010/63/EU on the protection of animals used for scientific purposes. They were approved by the Ethic committee for the animal experimentation of Rennes and received the approval of French minister of national education, research and innovation under the authorization number APAFIS# 15361-201806050954214. The authors followed the ARRIVE guidelines 2.0 for the design, analysis, and reporting of scientific research.

## Author contributions

VC, LC, and LL conceived the study. VB and TL carried out the experiments. VB and VC analyzed the data and drafted the manuscript. VC supervised the research. All authors contributed to the article and approved the submitted version.

## Funding

This research received the financial support of the PHASE research Department (INRAE).

## Conflict of interest

The authors declare that the research was conducted in the absence of any commercial or financial relationships that could be construed as a potential conflict of interest.

## Publisher’s note

All claims expressed in this article are solely those of the authors and do not necessarily represent those of their affiliated organizations, or those of the publisher, the editors and the reviewers. Any product that may be evaluated in this article, or claim that may be made by its manufacturer, is not guaranteed or endorsed by the publisher.
