# Supplementary material for: Environmental enrichment improves cognitive flexibility in rainbow trout in a visual discrimination task: first insights
Source: Front Vet Sci. 2023 Jun 15;10:1184296. doi: 10.3389/fvets.2023.1184296 (PMC10313407; doi:10.3389/fvets.2023.1184296)
Supplement: Supplementary file 1 [file Image_1.pdf]

Figure S1. Percentage of correct response per session for all the tasks for fish in early-Barren (A: #0624; B: #0647; C: #0977; D: #6228) and early-Enriched conditions (E: #6224; F: #6241; G: #6246; H: #6250). Each line colour corresponds to an exercise and dotted lines represent the transition from one exercise to another. Fish #0977 (C) has been stopped because of a technical issue, and fish #0647 (B) and #6241 (F) have been stopped because they reached the exclusion criteria.

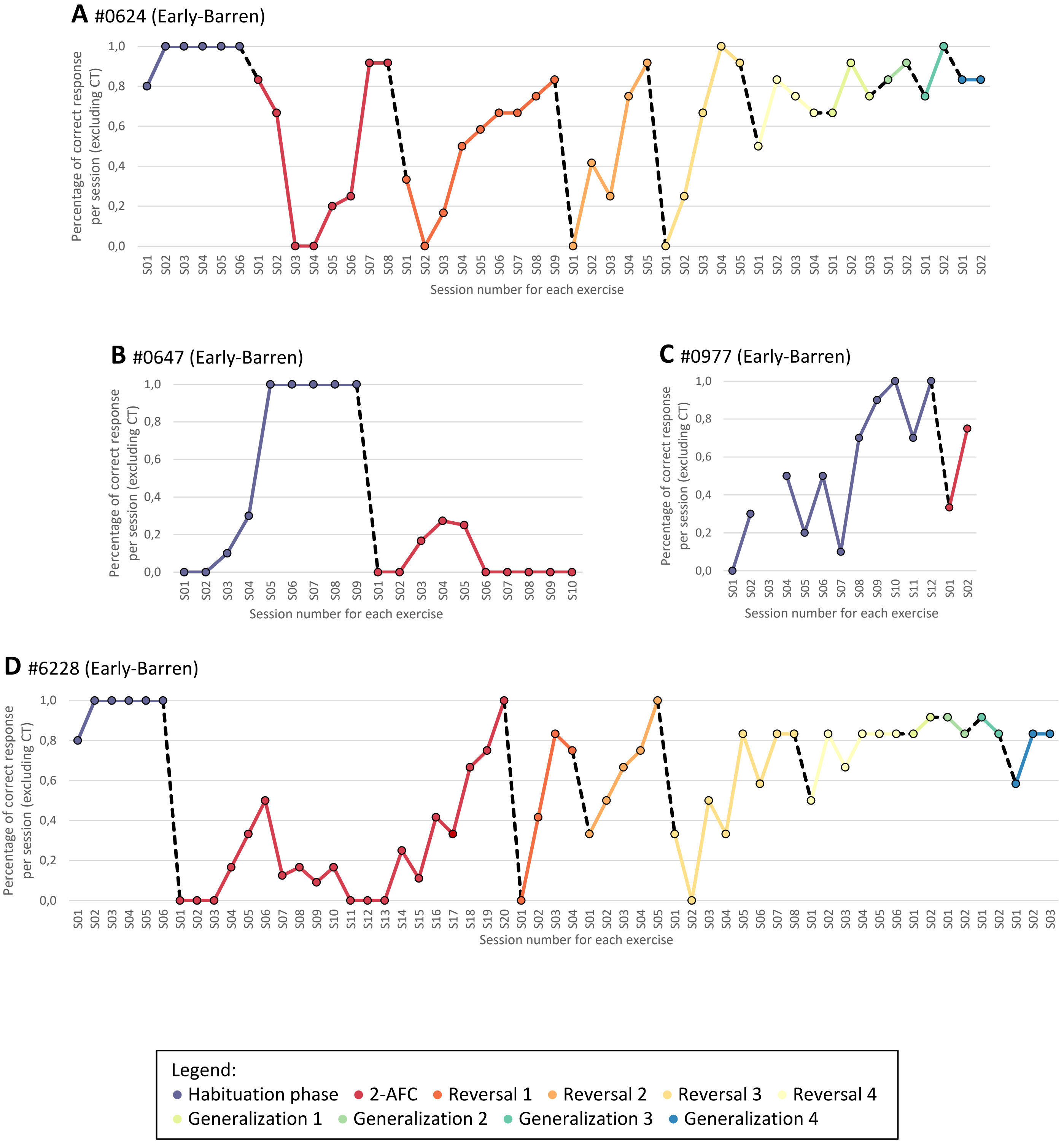

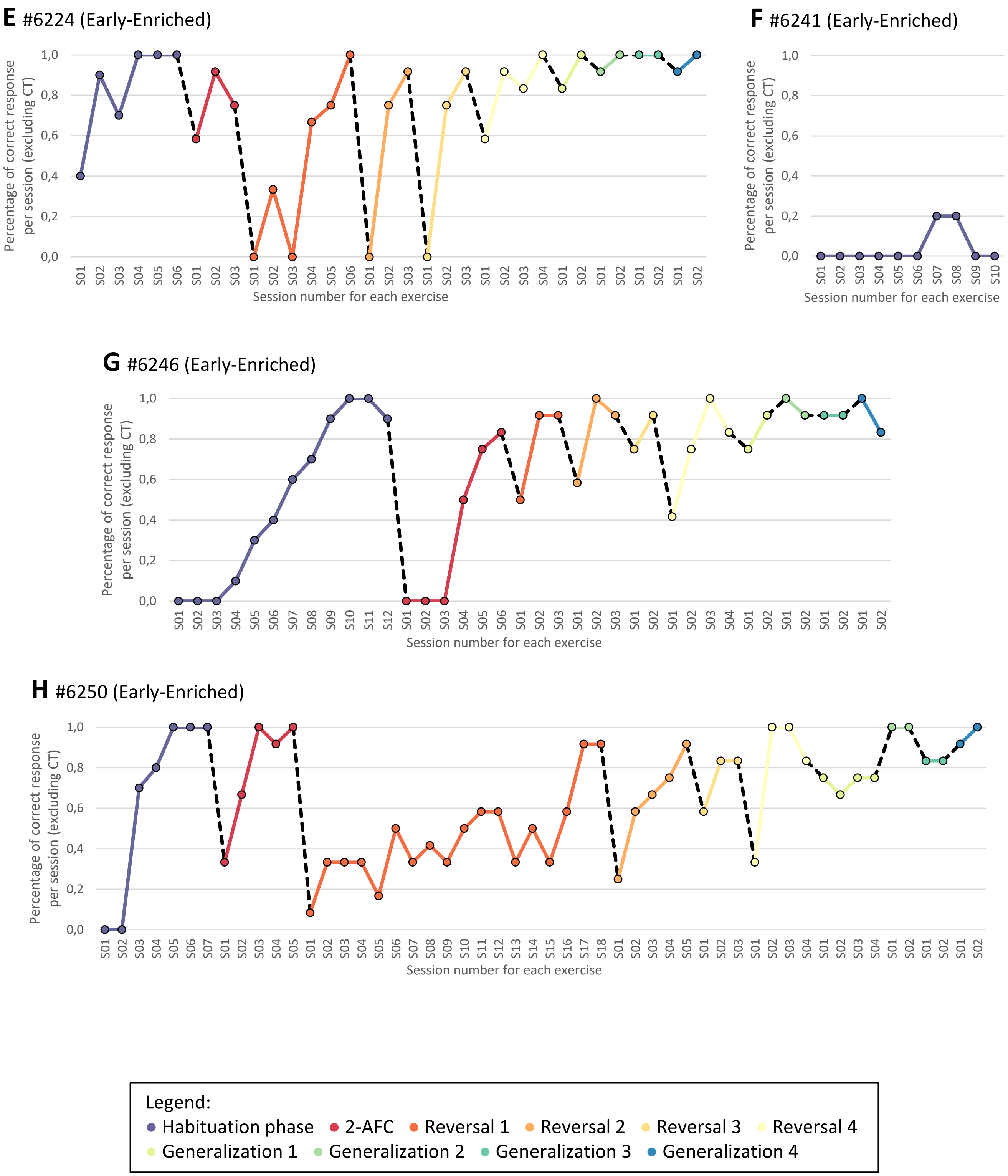
E #6224 (Early-Enriched)
Percentage of correct response per session (excluding CT)
1,0 0,8 0,6 0,4 0,2 0,0
S01 S02 S03 S04 S05 S06 S01 S02 S03 S01 S02 S03 S04 S05 S06 S01 S02 S03 S01 S02 S03 S01 S02 S03 S04 S01 S02 S01 S02 S01 S02 S01 S02
Session number for each exercise
F #6241 (Early-Enriched)
Percentage of correct response per session (excluding CT)
1,0 0,8 0,6 0,4 0,2 0,0
S01 S02 S03 S04 S05 S06 S07 S08 S09 S10
Session number for each exercise
G #6246 (Early-Enriched)
Percentage of correct response per session (excluding CT)
1,0 0,8 0,6 0,4 0,2 0,0
S01 S02 S03 S04 S05 S06 S07 S08 S09 S10 S11 S12 S01 S02 S03 S04 S05 S06 S01 S02 S03 S01 S02 S03 S01 S02 S01 S02 S03 S04 S01 S02 S01 S02 S01 S02 S01 S02
Session number for each exercise
H #6250 (Early-Enriched)
Percentage of correct response per session (excluding CT)
1,0 0,8 0,6 0,4 0,2 0,0
S01 S02 S03 S04 S05 S06 S07 S01 S02 S03 S04 S05 S01 S02 S03 S04 S05 S06 S07 S08 S09 S10 S11 S12 S13 S14 S15 S16 S17 S18 S01 S02 S03 S04 S05 S01 S02 S03 S01 S02 S03 S04 S01 S02 S03 S04 S01 S02 S01 S02 S01 S02
Session number for each exercise
Legend:
Habituation phase
2-AFC
Reversal 1
Reversal 2
Reversal 3
Reversal 4
Generalization 1
Generalization 2
Generalization 3
Generalization 4
